# Lectures on the Exploration and Treatment of Diseases of the Chest

**Published:** 1840-05-30

**Authors:** W. W. Gerhard


### MEDICAL EXAMINER.

DEVOTED TO MEDICINE, SURGERY, AND THE COLLATERAL SCIENCES.

No. 22.] PHILADELPHIA, SATURDAY, MAY 30, 1840. [Vol. III.

### LECTURES ON THE EXPLORATION AND TREATMENT OF DISEASES OF THE CHEST.

BY W. W. GERHARD, M. D.

LECTURE IV. — *Percussion—Rationale—Mode of performing—Pleximeter—Division of Chest into Regions—Value of Percussion.*

We now come to a highly important part of the subject—this is, percussion, or the method of estimating the density of the viscera contained within the thorax, by tapping lightly upon its surface. The rationale of this is very simple: The lungs occupy the greater part of the thoracic cavity, and are filled with air. If percussion be made upon them when removed from the body, they yield a very clear sound, especially if a solid yet elastic body be laid upon them, which may receive the impulsion of the striking body, and prevent it from sinking into the soft pulmonary tissue. Now, this elastic body, or sounding-board, exists naturally in the thorax, and is formed by ribs and cartilages; and a light tap upon their surface, that is, on the exterior of the chest, gives a clear, full, hollow sound. When the patient is thin, and the skin is very sensitive, he will not bear a smart tap without inconvenience; and, on the other hand, if he is very corpulent, or if the subcutaneous cellular tissue be infiltrated with serum, the sound will be quite dull, and will not truly represent the condition of the internal organs. In order to prevent this chance of error to the observer, and inconvenience to the patient, we interpose an additional elastic body between the chest and the end of our finger. This interposed body is called a pleximeter, and was invented by Dr. Piorry. Its only utility is to increase the body of sound, by giving more resonance to the elastic parietes of the chest, and to prevent the direct impression of the fingers upon the chest of the patient. Though the ribs are an excellent natural pleximeter, they are too sensitive at times, and at others are rendered useless for physical exploration, by the softer subcutaneous deposits. By applying an artificial pleximeter we not only increase the fitness of the natural sounding-board, if we may so call it, but we bring it into play, by compressing, and, as it were, thrusting out of the way the tissues which impede its vibrations, and then we gain the important advantage for ourselves and our patients of preventing pain, or perhaps mischief to the disease. The only method of performing percussion which is now practised, is that by means of the pleximeter. It has so many advantages over immediate percussion, or the striking with the ends of the fingers directly upon the chest, that it is much better fitted for every purpose.

The pleximeters used are various; that is, those that may be used; for, practically, they are reduced nearly to the most natural pleximeter, that is, the forefinger of the left hand. But if you choose, you may make use of a piece of gum elastic, of ivory, or of metal. You take this in the fingers of the left hand, and hold it firmly upon the chest, afterwards percussing in the usual way with the right hand. If it be not applied firmly against the chest, a clacking sound is immediately produced by the air which is interposed between the instrument and the skin: this clack cannot be entirely obviated, for the tap upon the instrument will of course give rise to sound. If the material be very dense, the sound will be sharp and decided, and interfere a little with the pulmonary sound, that is, the resonance developed by the tissue of the lungs; for this reason there are some advantages attending the use of the gum elastic pleximeter, rather than an ivory or metallic one, which is harder, and of course gives rise to more sound. The elastic instrument was, I believe, first proposed by Dr. J. B. S. Jackson, of Boston, and is the most convenient. You can readily enough make one for yourselves, by taking a common piece of gum elastic of the flat kind, about a quarter or a third of an inch thick, and about two inches square, that is of a size convenient for holding in the fingers. The density of gum elastic is more nearly similar to that of the chest, than a harder material, which is an additional reason for its employment, as it contributes to give it a clear, uncomplicated sound.

The gum elastic pleximeter is simple, but all of you are provided with one which is much more simple, and which you see me resort to—it is the forefinger of the left hand. In thin persons, the finger is even more bony and elastic than the ribs; in those who are fatter, or whose hand is remarkably stout and covered with a thick skin, the finger loses its elasticity, and is not so well fitted for the office of pleximeter. Still, under ordinary circumstances, it is the best one which you can employ, and is superior to any of the ordinary artificial instruments, from its ready adaptation to different parts and irregularities in the chest. The finger may in this way be placed behind the clavicle or below it, and be brought very near the lung, which could not be done with a broad and flat plate: any single limited spot may be examined in the same way with great ease. This natural pleximeter may be used in two ways: you may apply the dorsal or palmar surface upon the chest, and of course tap upon the reversed side; if you apply the palmar surface upon the chest, the dorsal side upon which the percussion is made is firm, and gives a sharp clear sound; it is much better, therefore, for the accurate appreciation of slight deviations from the natural standard. The palmar surface is occasionally more convenient, especially when it becomes necessary to apply the finger to the depression behind the clavicle,—it is of course better for this purpose that the finger should be curved to fit this depression; hence, percussion must be made upon the palmar surface. Much of this nicety in the mode of applying the finger which serves as a pleximeter, will be found to be unnecessary, and may be dispensed with after a little practice; the shape of the hand and fingers of the observer will, however, have some influence on the position which will be found in practice most convenient.

The most difficult part of percussion is not, however, the application of the hand which serves as a pleximeter; this is very soon acquired. Much difficulty, however, is often met with as to the method of tapping or striking with the right hand upon the pleximeter finger. You may use for this purpose either one finger or several, but you will find that for children, and for persons who are very thin, and whose chests are therefore tender, a single finger will be most convenient. Whether you use one or more fingers, the essential part of the process is to hold the hand as firm as possible, and to give the greatest possible elasticity to the wrist. The motion should therefore be performed in the wrist, and not in the shoulder or elbow; if you strike with the whole arm, however gently it may be, you are apt to give the patient pain, and you are sure to deaden the sound. The sound depends upon the elasticity of the wrist, and if the fingers be suffered to remain in contact with the pleximeter, or the thorax, a moment longer than is necessary for the percussion, the sound will be proportionately obscured.

The slowness of the motion is a frequent error with those who are slightly acquainted with physical exploration. They are apt to pause as soon as the finger touches the surface, and allow it to remain in contact with the part, this is altogether wrong. It is at first difficult to acquire the perfect freedom of motion which is essential to elastic, clear percussion; still, it is perfectly practicable, with a little perseverance and experience.

If you use a single finger for purposes of percussion, there is little difficulty in holding it in the proper position. Either the fore or the middle finger of the right hand may be selected as the percussor; you then bring it, as nearly as possible, into the form of a light mallet or hammer, and make the second and third phalanges serve as the head of the hammer; of course, they must be flexed at right angles with the first phalanx, and must be retained firmly in that position, otherwise the form of the hammer is lost. The extremity of the finger should be as nearly at right angles with the hand as possible, otherwise the tap is not made with the extremity of the fingers, but the pulp, which is a matter of essential consequence, as the pulp of the fingers is soft, and non-elastic, and deadens the sound. If the thorax be covered with fat, or the parietes be infiltrated, it is necessary to percuss more strongly than is possible with a single finger; in that case bring the three middle fingers of the hand together, and allow them to rebound together after striking upon the pleximeter; they thus give a more forcible impulsion, and a sound nearly as clear as if a single finger were used. Indeed, you will generally find this method the most convenient for the examination of the chest, although, as I have already stated, a single finger is the best percussor in cases of children whose chest is thin and very elastic, or in those whose thorax is very nearly in the condition of that of children, from emaciation. Although when you use several fingers, your tap is of course stronger than if a single one be employed, you will find in either case that it is not the force, but the sharpness and quickness of the impulsion, which produces the sound. A hard blow causes so much clacking sound against the finger that it proves a source of error, and renders the full resonance of the chest more difficult to draw out.

Plain and easy as these directions are, probably not one of you will at first practise them correctly; you will find that the elasticity of wrist, and light, clear tap, are learned but slowly, and after many efforts. There is, however, an easy method of improving your knowledge of percussion: repeat the operation frequently upon yourselves, at night, when you have removed your outer clothing, and all is quiet around you, a slight difference in sound then becomes perceptible, and the causes which render it dull are evident, and you thus learn to avoid those errors which are embarrassing from their apparent trifling insignificance. Notwithstanding all your care, you will not make equal progress in this matter; to acquire a perfect facility, a light and rather thin hand, and a correct ear, are requisite; if you have not these advantages, you will experience more difficulty,—but with more practice and more attention, it may be overcome.

An instrument has been contrived by Dr. Bigelow, of Boston, for percussion. It is a piece of whalebone or elastic wood, covered at the end with a ball of velvet or buckskin; the ball is nearly an inch in diameter: it is a very good instrument if any accident should deprive you of the full use of your fingers: the objections to it are, of course, the trouble and complexity of its use; hence Dr. Bigelow himself advises it merely in hospital practice, where you have a large number of patients to examine, and your fingers sometimes suffer from constant tapping. If you use this instrument, tap with the ball upon the pleximeter, which should be made of gum elastic.

While I was at Paris, an ingenious friend of mine imagined an instrument for measuring the sound of percussion. It was to consist of a percussor somewhat similar to that of Dr. Bigelow, but inclosed in a large stethoscope. The percussor was to be set in motion by a spring and wheel, as in watches, and the ear to be applied to the stethoscope in the usual way during the action of the instrument. The idea was ingenious, but the practical application of it almost impossible. Any contrivance to assist the senses in diagnosis must be extremely simple, or it will be practically useless; and, as a general rule, you will do much better to trust to your hands alone for the percussion of the chest.

Percussion is applicable to the study of abdominal as well as thoracic diseases; indeed, it is largely applicable to the exploration of many diseases of the former cavity. The abdomen contains solid viscera, such as the spleen and liver, and tubes filled with gas or liquid. The gaseous contents are much more abundant than the liquid; hence the sound is clear over the greater part of the abdomen from the gas retained in the alimentary canal. If the quantity of gas be increased, you necessarily have an increased resonance on percussion, and the converse is, of course, true; this fact enables you to estimate the effusion of liquid in the peritoneum, the enlargement of the solid viscera, and the distension of the cavity of the intestine with a large quantity of gas, which causes a tympanitic resonance. The same manual method of percussion is applicable here as in the exploration of the thorax; but, in general, you will find that a very light tap, with a single finger, is the best, especially in those cases in which the gas is contained in the larger intestines, and therefore approaches very near to the surface.

Percussion of the abdomen is always practised when the patient is lying upon his back, and the surface of the abdomen therefore placed in the situation most convenient for examination; but in the thorax you vary the position,—that is, you vary it in all those cases in which the patient is well enough to change his posture at pleasure: if he be too feeble for this, you must, of course, examine him in any way that happens to be practicable. In ordinary percussion your object is to place the patient in such a position that you may render the parietes of the chest as tense, and consequently as elastic as possible; the muscles must therefore be put upon the stretch, and the skin drawn tightly backwards. In percussing the anterior part of the chest, the patient should sit upon a chair; or, which is still better, stand erect, and throw the shoulders slightly backwards, so as to render the pectoral muscles tense; for the posterior part of the chest the position should be reversed; the patient must lean forward, and cross his arms strongly, to draw the scapulæ from the spine, and throw out the arch of the back. To examine the axillary region, the arms should be raised above the head. The chest may be percussed at first in a cursory way on each side, to gain a general idea of the condition of the viscera, and afterwards you may proceed to the details, and compare the sonorousness of different parts of the lungs and of the heart. The lungs are not equally sonorous throughout their whole extent; for as the clearness of the sound depends upon the large quantity of air contained in the vesicles, and the small quantity of solid matter, a difference in the relative proportions of these parts will give rise to various degrees of resonance; thus, the sound is most clear wherever the vesicles are most numerous, and the larger bronchial tubes, whose walls are thick and firm, are least developed; for the thin parietes of the vesicles present no obstruction to the vibration of the air contained within them, but the hard walls of the bronchial tubes offer a very decided obstacle. Hence, if other things be equal, the sound may be stated to be most clear at the lower part of the chest, and along the anterior margin of the lungs, while it is comparatively dull at the summit and root; in the rest of the lungs the sound is intermediate, neither dull nor clear. Where the lungs are so situated as to overlap the more solid organs of viscera contained in the chest, the sound is but moderately clear, becoming more dull as the thickness of the solid organ is greater than that of the lungs. This is the case both with the liver and heart, and is a fact which is analogous to the phenomena observed in a diseased state of the lungs, where a lesion which renders the deeply seated parts of the pulmonary tissue more solid, makes the percussion dull over the corresponding parts of the lung. The dulness of sound is observed, notwithstanding the superficial portions may be perfectly pervious to the air.

The relative quantity of bronchial and vesicular tissue gives rise to the modifications in the clearness of the sound in percussion to which I have alluded, and the resonance of the vesicular structure is quite different from that which would be caused by the same quantity of air contained in a single bag, or large vesicle. If the air contained in a large number of scattered vesicles were collected together, and percussion were made upon the sac which contains it, the sound would be drum-like, or tympanitic. This character is actually observed in certain morbid conditions of the chest, but is never similar to the healthy sound, which is more deep and hollow, but at the same time less gaseous. The difference between the two varieties of the clear sound will be appreciated at once if you examine the chest, and then percuss downwards until you come to the hollow viscera of the abdomen, which yield the tympanitic resonance very different from the hollow sound, which you may call vesicular.

After you have gained a general idea of the resonance of the chest, you should proceed to a more thorough examination of the various portions of it, one by one. For this purpose, it is convenient to divide the chest into regions or parts. These may be the anatomical divisions corresponding to the exterior of the chest, as the clavicular, scapular regions, &c.; or you may use terms expressive merely of the fractional parts into which the surface is divided, such as thirds, fourths, &c. For most purposes, the latter method has seemed to me to be the most convenient. When you wish to be more exact, you may subdivide these regions, or you may, in addition, designate them by a reference to their anatomical relations; but if you divide the anterior and posterior surfaces into three parts, and the axillary into two, you will find it sufficiently minute for most purposes. The anterior surface may be divided, therefore, first, into an upper third, extending from the summit of the lung to the lower margin of the second rib, and of course including the anatomical subdivisions of post-clavicular, or the space above the clavicle; clavicular, or that corresponding directly to this bone; and sub-clavicular, or the region found immediately beneath it. This portion, in general terms, may be said to correspond with the summit of the lung, and is of great interest to the physician; for it is the ordinary seat of tuberculous diseases, which of course render the sound dull; and occasionally of pneumonia, which produces the same effect in a more marked degree; and, thirdly, of emphysema, which renders the sound preternaturally clear. The middle third extends from the lower margin of the upper division to the space between the fourth and fifth ribs; it is less interesting for practical study, for its diseases are, for the most part, such as begin in the upper or in the lower third, and extend themselves to the middle, than those which commence in it. Emphysema, however, is often more developed about the middle of the lung than in any other part of this surface. The lower third extends from the boundary of the second to the lower margin of the chest; it is the usual seat of pleuritic effusions and of hydrothorax; in both of these diseases the liquid extends itself gradually from the posterior parts of the chest, towards its anterior margin, rendering the lower portion dull.

In the healthy condition the sounds of percussion are not equally sonorous in all parts of the anterior part of the chest; in children the lower third is decidedly the most sonorous; in adults the middle is generally the clearest. In women you will find it difficult to compare these various portions together, for the mammæ interfere so much with percussion that it is extremely difficult to examine the middle third in a satisfactory way. The heart is another cause of dulness of sound at the interior part of the lower third on the left side. The præcordial dulness extends from the space between the fourth and fifth ribs at the sternum to the nipple, generally passing a little within this part. On the right the dulness is bounded by a line which follows the middle of the sternum; the lower part of the heart rests upon the diaphragm.

The axillary or middle surfaces are divided most conveniently into two portions, by a line drawn nearly through the middle of the axillæ. The sound in these parts differs in a very slight degree, and is throughout extremely clear, from the almost complete absence of the more solid parts of the lungs, and the remarkable freedom of this portion of the chest from muscles which necessarily deaden the sound to a greater or less degree.

If the posterior part of the chest be divided into thirds, these portions are still more unequal than they are at the anterior part. The upper extends from the top of the lungs to a line passing along the spine of the scapula, prolonged to the vertebræ. This, like the summit of the lungs at its anterior part, is the common seat of tubercles, which are more frequently developed here than at any other portion. Percussion is, however, so difficult at this part of the lungs from the thickness of the muscles, that its results are not of great value to beginners. Under all circumstances the sound is but moderately clear, becoming duller towards the external margin. The middle third extends from the lower margin of the upper, to a line drawn at right angles to the spine from the lower angle of the scapula. The natural sound is here much more clear than in the upper third, especially near the spine, where the scapula does not interfere with it. Upon the scapula the percussion is necessarily dull. The lower third corresponds to the largest mass of pulmonary tissue; and, from the conformation of the ribs, gives a remarkably clear sound in children, whose thorax is elastic. In adults, the greater firmness of the ribs and muscles, and the greater induration of the ligamentous and cartilaginous tissue, renders this sound less hollow; still it is always comparatively clear. This portion of the chest, with the middle third, is the usual seat of pneumonia; it is also the commencing point of pleuritic effusions,—hence, in disease it is often dull, when the rest of the chest is comparatively clear.

After you have examined the chest in a cursory manner, the regions must be examined comparatively,—that is, each part should be compared with the corresponding one upon the opposite side at the same points. For purposes of convenience I generally begin at the summit of the lung, at the anterior part, and then pass downwards towards the diaphragm, percussing both over the ribs and in the intercostal spaces, and always placing the finger of the left hand parallel to the ribs; this gives you the sound corresponding accurately with the portion of lung which is immediately beneath your finger, or very little more than the sound corresponding with that space. If you percuss across several ribs, the sound is more difficult to appreciate, as it is produced by a much larger portion of the lung, and is therefore of little value, except for the facility which it gives you of gaining a general idea of the condition of the lungs. If you are at all doubtful about the sound, I would advise you always to compare the two sides together in very quick succession, while the impression of the sound is still fresh in your senses, and repeat the percussion until you are satisfied whether there is, or is not a real difference.

In a certain proportion of diseases of the lungs, the signs of percussion, united with the general symptoms, are sufficient for the diagnosis; or, if combined with the other physical signs, they are sometimes perfectly characteristic of the disease without the aid of the rational symptoms. You must remember, however, that percussion indicates merely the relative density of the lung, and is not sufficient for the diagnosis of most of its diseases without the aid of other means of investigation. The signs of percussion, although comparatively few in number, are often of more value than any others, for their evidence is positive as far as it is applicable, and indicates with perfect accuracy the density of the tissue beneath the spot upon which the percussion is made; but as the causes which influence the density are numerous, they are not explicable without the comparison of other symptoms. Percussion is, therefore, of all the signs of pulmonary disease, the most strictly physical, and of course the most mathematically correct. Percussion is not confined to the diseases of the lungs; for as these organs surround the heart, the sound is clear as far as their tissue extends: hence, the size of the heart is measured by percussion of the lungs, rather than of the organ itself. It is, as we shall afterwards see, one of the most certain methods of learning the size of the heart.

The practical mode of acquiring percussion is of more interest to you than the mere detail of the signs derived from it. Like all the means of pectoral investigation, percussion may be learned in two ways,—that is, either on the healthy or the diseased subject. Those of you who observe patients on a large scale, and have sufficient time to examine at your own leisure the cases which I point out to you, will learn percussion chiefly from patients, and, as it were, in connection with other signs. But this is not always the more convenient method: it is not at all fitted for those whose sense of hearing is not acute, or who may not possess the necessary facilities for studying disease among a large collection of patients. If your ear is to be educated as well as your hand, you will cause no little uneasiness to your patients in your attempts to gain, little by little, a familiarity with the sounds. You will be sure to percuss much too smartly for their comfort at least, and you may possibly aggravate the symptoms of their diseases. Always, therefore, learn on your own persons; or if several of you unite together, and form little clubs for mutual percussion, you will get on much more rapidly. For the healthy chest presents every shade of percussion, from complete flatness to the most perfect sonorousness, and you may thus accustom yourselves to every variety of sound. At first you should examine the parts of the chest where the sounds are most distinct; and for this purpose it is best to select a young person, and, if possible, one who is rather thin,—then, by percussing first on the middle of the side of the chest near the sternum, and afterwards on the region of the liver, you may gain a correct idea of the difference between perfect flatness, and the full, clear, pulmonary sound. This should be repeated frequently, until a good idea of the difference of these sounds is impressed upon the memory, and, above all, upon the senses. The same points of extreme flatness and sonorousness will explain the difference between the tapping with a single finger, and the deeper, but less sharp sound produced by decided percussion with several fingers. These comparative points should be examined on several individuals of different ages, and different degrees of flatness or thinness, until a correct idea of the average sounds is acquired. After the extreme degrees of sound have been repeatedly heard, the intermediate characters may be learned by percussion of the præcordial region, where the sound is dull, but in the healthy subject not completely flat. There is also a little dulness of sound at the summit of the lungs; on the right side, in most individuals, it is a little less clear than upon the left. The repeated examination of these parts of the chest will not only give you a correct idea of the sounds themselves, but will train your ear and hand to the manual performance of percussion.

I have pointed out to you the great accuracy of the signs of percussion, and their uniform dependence upon the same physical condition of the lungs. It matters but little whether the disease is seated on the surface of the lung or in the internal parts of it; the quantity of air is necessarily diminished by every hardening of the tissue, which is sufficiently extensive to compress one or more lobules. Whenever the obstruction is sufficient to form an alteration in the sound perceptible to our senses, it may be readily recognised. The induration is extensive enough when three or four lobules become impervious to the air, but it cannot be recognised with certainty if limited to a less extent. The deeper seated lesions are rather more obscure than those nearer the surface, as the air-vesicles which intervene between the ear and the indurated portion, of course give rise to a clear sound, but it is less full and hollow than it is when the lung is completely free; for the plain reason that the mass of sonorous, that is, of aerated tissue, beneath your finger, is less considerable.

A compression of the lung necessarily acts much in the same way as an induration of its parenchyma; hence effusions into the pleuræ, or even into the pericardium, compress the pulmonary tissue, and render it less elastic,—that is, they diminish the size, and expel the contents of the air-cells. The compression which is at first produced does not give rise to as great a degree of dulness as the induration of the pulmonary tissue, for the whole tissue remains, and is merely a little less distended with air than usual; but in advanced cases of effusions into the pleura the flatness may be more complete than under any other circumstances, for the compression, although slow, may be carried to such a point as to alter the structure of the pulmonary tissue completely, and flatten it against the spine. In the pericardial effusions the compression is never so great as to destroy the resonance, except immediately around the liquid.

The condition of life has nothing to do with the clearness or dulness on percussion,—for in the lung removed from the dead body you will find precisely the same condition of things under the same circumstances,—and you may readily verify the fact for yourselves, if you attempt to make the examination of the body of an individual dead of a disease which alters the structure of the lung,—or you may resort to the same observation, by producing a change in the structure of the lung by artificial means, such as injections of wax into the bronchial tubes, or of liquids or of air into the serous cavities; if, on the other hand, you distend the vesicles by inflating them with air, the percussion immediately becomes extremely resonant.

You will find that in healthy individuals there is often a considerable difference in the sounds of percussion. I have already alluded to some of the causes of this difference, which may be perfectly external to the chest, and consist in accumulations of fat or serum beneath the skin; or, on the other hand, they may depend upon a want of resonance in the thoracic parietes, and arise from the partial ossification of the cartilages. There is a third class of patients who offer less than the average degree of resonance of the chest; in these individuals the lungs contain less air than usual, and are apparently more firm and more similar to cellular tissue. The chest, on the other hand, may be more resonant than usual, from either a real dilatation of the vesicles of the lungs, or from the patient being greatly emaciated, without much disease of the lungs themselves. You will find but one way of overcoming these difficulties,—and that is, to examine the chest in many patients until you acquire a knowledge of the average clearness or dulness of sound, and of the circumstances which modify it without the existence of positive disease of the lungs. These accidental circumstances are altogether dependent upon the ordinary acoustic principles, elasticity and thinness of the parietes of the chest favouring the clearness of sound, and thickness and rigidity of them producing a contrary effect.

